# Hsa_circ_0070963 inhibits liver fibrosis via regulation of miR-223-3p and LEMD3

**DOI:** 10.18632/aging.102705

**Published:** 2020-01-29

**Authors:** Dong Ji, Guo-Feng Chen, Jin-Cheng Wang, Si-Han Ji, Xue-Wen Wu, Xiao-Jie Lu, Jin-Lian Chen, Jing-Tao Li

**Affiliations:** 1 Second Liver Cirrhosis Diagnosis and Treatment Center, The Fifth Medical Center of Chinese PLA General Hospital, Beijing, China; 2 Department of General Surgery, The First Affiliated Hospital of Nanjing Medical University, Nanjing, China; 3 Sparkfire Scientific Research Group of Nanjing Medical University, Nanjing, China; 4 Department of Gastroenterology, Fengxian Hospital, Southern Medical University, Shanghai, China; 5 Department of Gastroenterology, Shanghai Sixth People’s Hospital (South), Shanghai Jiaotong University, Shanghai, China; 6 Department of Liver Diseases, The Affiliated Hospital of Shaanxi University of Chinese Medicine, Xianyang, Shaanxi Province, China

**Keywords:** hsa_circ_0070963, miR-223-3p, LEMD3, hepatic stellate cells, liver fibrosis

## Abstract

Previous circular RNA (circRNA) microarray analyses have uncovered an abnormal expression of hsa_circ_0070963 in hepatic stellate cells (HSCs). However, the specific role of hsa_circ_0070963 in liver fibrosis remains unknown. Here, we show that hsa_circ_0070963 inhibits liver fibrosis via regulation of miR-223-3p and LEMD3. Moreover, we demonstrated that hsa_circ_0070963 levels were reduced during liver fibrosis while restoring hsa_circ_0070963 levels abolished HSC activation, with a reduction in α-SMA and type I collagen levels both *in vitro* and *in vivo*. Furthermore, hsa_circ_0070963 overexpression suppressed both cell proliferation and the cell cycle of HSCs. MiR-223-3p was confirmed as a target of hsa_circ_0070963 and was shown to be involved in the effects of hsa_circ_0070963 on HSC activation. Furthermore, LEMD3 was confirmed as a target of miR-223-3p and was shown to be responsible for the activation of HSCs. The interactions between hsa_circ_0070963, miR-223-3p, and LEMD3 were validated via bioinformatic analysis, luciferase reporter assays, and rescue experiments. Collectively, hsa_circ_0070963 appeared to function as a miR-223-3p sponge that inhibited HSC activation in liver fibrosis via regulation of miR-223-3p and LEMD3. Therefore, hsa_circ_0070963 may serve as a potential therapeutic target for liver fibrosis.

## INTRODUCTION

Liver fibrosis is characterized by a complicated process involving both parenchymal and non-parenchymal liver cells, as well as infiltrating immune cells. Moreover, it serves as a common stage between the development of various chronic liver diseases and cirrhosis. However, cirrhosis is the leading cause of morbidity and mortality worldwide [1], resulting in an unsolvable medical problem. Liver fibrosis is characterized by the excessive accumulation of extracellular matrix (ECM) proteins such as collagen type I and III [2] in the liver, which eventually leads to fibrotic tissue deposition and hepatic dysfunction. In the fibrotic liver, hepatic stellate cells (HSCs) become activated and undergo myofibroblastic transdifferentiation [3–5]. These myofibroblastic HSCs are mainly responsible for the production of ECM proteins. Therefore, inhibition of activated HSCs is a good therapeutic strategy to combat liver fibrosis.

Circular RNAs (circRNAs) are a newly discovered class of non-coding RNAs (ncRNAs), which are characterized by a covalently closed structure lacking the 5′ to 3′ polarity or polyadenylated tail [6]. While different from traditional linear RNAs, circRNAs are abundant in the cytoplasm of mammalian cells and show high species-specific, tissue-specific, and disease-specific expression [7]. Due to their specific structure, circRNAs are more resistant to RNA exonucleases and therefore have improved stability [8]. By sponging microRNAs (miRNAs) or interacting with other molecules, circRNAs regulate gene expression at both the transcription and translational level [9]. Moreover, accumulating evidence suggests that circRNAs are involved in the development of several cancers, such as hepatocellular carcinoma (HCC), colorectal cancer, gastric cancer, and lung cancer [10–13]. As a result, circRNAs are considered to be suitable as biomarkers or prognosis factors in various diseases. The expression pattern of circRNAs in liver fibrosis has been previously reported [14, 15]. We found that hsa_circ_0070963 expression was abnormally downregulated in irradiated HSCs. However, the potential role of hsa_circ_0070963 in the process of liver fibrosis remains unclear. By using the TargetScan and miRanda database, we found seed matches between hsa_circ_0070963 and miR-223-3p. Moreover, by using miRTarBase, we found that LEMD3 is one of the target genes of miR-223-3p. A recent study showed that miR-223-3p is a novel anti-inflammatory and anti-fibrotic therapeutic target [16]. Furthermore, the LEMD3 protein is known to act as an antagonist to transforming growth factor-beta (TGF-β) signaling at the inner nuclear membrane and is associated with many diseases. Therefore, it could conceivably be speculated that hsa_circ_0070963 and LEMD3 mRNA may act as a pair of competitive endogenous RNAs (ceRNAs) that are linked by miR-223-3p.

In this study, we examined the expression of hsa_circ_0070963 and its biological significance in liver fibrosis. We speculated that hsa_circ_0070963 might be involved in HSC activation by affecting the expression of LEMD3 in a miR-223-3p-mediated manner. Cellular and molecular biology experiments *in vitro* or *in vivo* were used to verify this hypothesis. As such, this study provides novel evidence that may be used to develop effective anti-fibrotic treatment strategies in the clinic.

## RESULTS

### Hsa_circ_0070963 is downregulated during liver fibrosis

**Figure 1:**
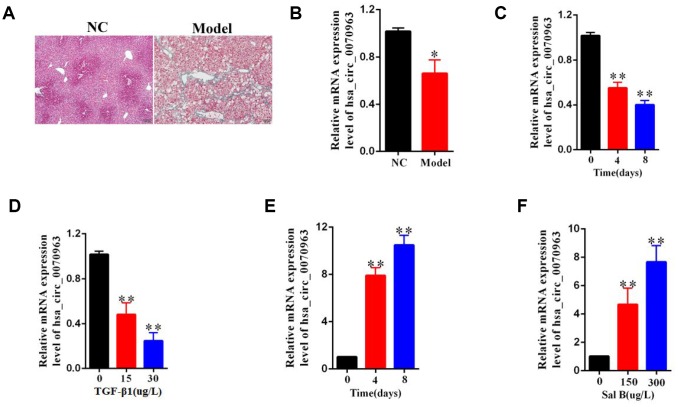
**Downregulation of hsa_circ_0070963 in liver fibrosis.** (**A**) Liver fibrosis was confirmed by Masson staining in CCl_4_-treated mice. The scale bar represents 100 μm. (**B**) Hsa_circ_0070963 expression was analyzed in primary HSCs isolated from NC or CCl_4_-treated mice. (**C**) Relative hsa_circ_0070963 gene expression was detected in the TGF-β1-treated LX2 cell line during culture days. (**D**) Hsa_circ_0070963 expression was examined in the LX2 cell line treated with increasing concentrations of TGF-β1. (**E**) Relative hsa_circ_0070963 gene expression was detected in the Sal B-treated LX2 cell line during culture days. (**F**) Hsa_circ_0070963 expression was examined in the LX2 cell line treated with increasing concentrations of Sal B. Data are presented as means ± SD of three experiments (^*^p < 0.05 and ^**^p < 0.01).

Among mouse models, carbon tetrachloride (CCl_4_) is the most commonly used hepatotoxic reagent to produce a typical liver fibrosis model [17]. As shown by the Masson staining, the collagen expression was used to confirm the occurrence of liver fibrosis (Figure 1A). It is known that freshly isolated HSCs are activated during the first days of culturing, with a reduction in quiescent phenotype markers and an increase in mesenchymal phenotype markers [18]. Next, primary HSCs were isolated from both the control mice and the CCl_4_-treated mice. Compared with the quiescent HSCs from the control mice, qRT-PCR analysis showed that hsa_circ_0070963 expression was reduced in the activated HSCs from CCl_4_-treated mice (Figure 1B). Then, we stimulated the human HSC cell line LX2 with transforming growth factor β1 (TGF-β1) and found that hsa_circ_0070963 expression levels were significantly lower than those in the control group (Figure 1C, 1D). Sal B has been reported to suppress HSC proliferation, collagen production, and HSC transdifferentiation *in vitro* [19, 20]. Our results suggested that the expression of hsa_circ_0070963 was significantly enhanced by Sal B in a time-dependent and dose-dependent manner (Figure 1E, 1F). Overall, these results indicate that hsa_circ_0070963 levels are reduced during liver fibrosis and HSC activation.

### Upregulation of hsa_circ_0070963 suppresses activation of HSCs *in vitro*

**Figure 2:**
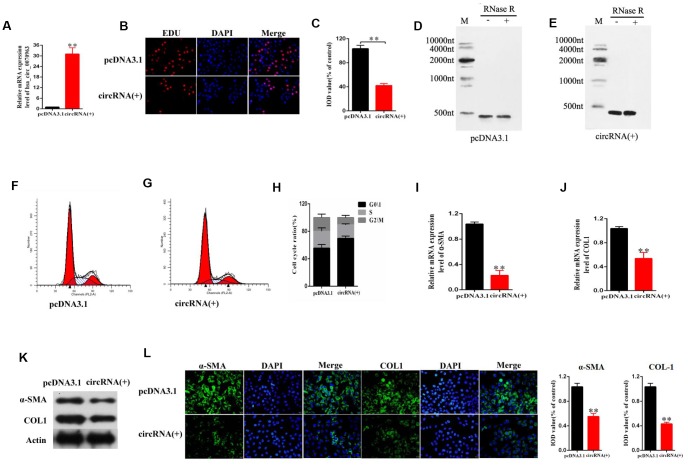
**Effects of hsa_circ_0070963 overexpression on HSCs.** LX2 cells were transduced with hsa_circ_0070963 for 48 h. (**A**) hsa_circ_0070963 mRNA levels in transduced LX2 cells. (**B**, **C**) HSC proliferation was detected using the 5-Ethynyl-20-deoxyuridine (EdU) assay (**B**) and the IOD values are shown (**C**). (**D**, **E**) Northern blot analysis of hsa_circ_0070963 expression after RNase R treatment. (**F**–**H**) Cell cycle analysis was performed with control or hsa_circ_0070963-overexpressing cells and the cell cycle distribution is shown. (**I**, **J**) The mRNA levels of α-SMA (**G**) and Col1A1 (**H**) were measured by qRT-PCR. (**K**) The protein levels of α-SMA and Col1A1 were measured by western blot. (**L**) Immunofluorescence staining for α-SMA (green) and Col1A1 (green) was evaluated by confocal laser microscopy. DAPI was used to stain the nuclei. The scale bar represents 50 μm. Data are presented as means ± SD of three experiments (^*^p < 0.05 and ^**^p < 0.01).

To assess the specific role of hsa_circ_0070963 in HSC activation, the human HSC cell line LX2 was transfected with hsa_circ_0070963 and its expression was examined by qRT-PCR. As expected, our results showed an obvious increase in hsa_circ_0070963 levels (Figure 2A). Using EdU assays, we showed that HSC proliferation was suppressed by hsa_circ_0070963 overexpression (Figure 2B, 2C). Northern blot for hsa_circ_0070963 treated with and without RNase R was performed. As shown in Figure 2D and 2E, hsa_circ_0070963 was successfully overexpressed in an annular form. Next, we determined that most of the cells were distributed in the G1 phase after upregulation of hsa_circ_0070963 by cell cycle analysis, which suggested that hsa_circ_0070963 induced G1/S phase cell cycle arrest (Figure 2F–2H). Moreover, the mRNA level (Figure 2I, 2J) and protein level (Figure 2K) of α-SMA and type I collagen (ColA1) were remarkably decreased in the presence of hsa_circ_0070963. This was confirmed by immunofluorescence analysis, which indicated that hsa_circ_0070963 overexpression resulted in a decrease in α-SMA and ColA1 (Figure 2L). These data provide compelling evidence of the inhibitory role of hsa_circ_0070963 in HSC activation.

### Hsa_circ_0070963 acts as a molecular sponge for miR-223-3p

**Figure 3:**
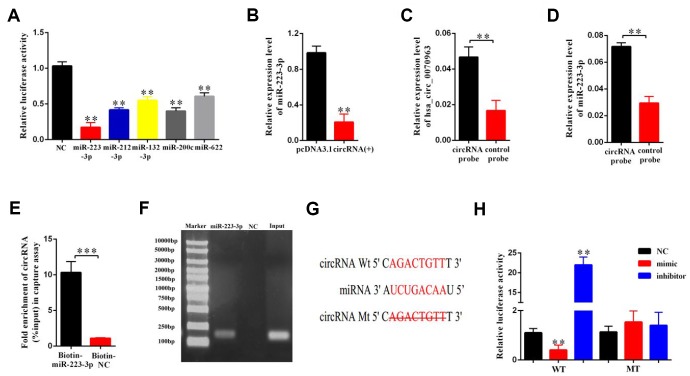
**miR-223-3p is a direct target of hsa_circ_0070963.** (**A**) The luciferase activity of hsa_circ_0070963 after transfection with pGL3-Basic-hsa_circ_0070963 combined with each miRNA mimic in HEK-293T cells. (**B**) The mRNA level of miR-223-3p was analyzed in hsa_circ_0070963-overexpressing LX2 cells. (**C**) Hsa_circ_0070963 in LX2 cell lysates was pulled down and enriched with an hsa_circ_0070963 specific probe and then detected by qRT-PCR. (**D**) MiR-223-3p in LX2 cell lysates was pulled down and enriched with an hsa_circ_0070963 specific probe and then detected by qRT-PCR. (**E**) Pull-down assay to validate the direct interaction between hsa_circ_0070963 and miR-223-3p. Biotin-coupled NC is not complementary to hsa_circ_0070963. (**F**) The product of (**D**) was detected using qRT-PCR, followed by agarose gel electrophoresis. (**G**) Predicted binding sites of miR-223-3p and hsa_circ_0070963 and mutated hsa_circ_0070963. (**H**) Relative luciferase activity was determined at 48 h after transfection with miR-223-3p NC/mimic/inhibitor or hsa_circ_0070963 wild-type (WT)/Mutant (Mu) in HEK293T cells. Data are presented as means ± SD of three experiments (^*^p < 0.05, ^**^p < 0.01, and ^***^p < 0.001).

CircRNAs have been reported to function as miRNA sponges to competitively bind miRNAs and regulate downstream gene expression [21]. Previously [14], we used bioinformatics analysis (TargetScan and miRanda database) to determine that hsa_circ_0070963 shares a complementary matching sequence of 5 miRNAs, which might bind with hsa_circ_0070963 in HSCs. As illustrated in Figure 3A and 3B, among the 5 miRNAs, miR-223-3p was most significantly decreased in hsa_circ_0070963-overexpressing cells, indicating a potential strong association between these two ncRNAs. As such, we performed a biotin-coupled probe pull-down assay to confirm this assumption. Compared with the control group, we detected a specific enrichment of hsa_circ_0070963 and miR-223-3p in the hsa_circ_0070963 pulled down pellet, suggesting that hsa_circ_0070963 could directly sponge miR-223-3p (Figure 3C, 3D). To further validate the sponge activity of hsa_circ_0070963, we performed a biotin-coupled miRNA capture. Similar to our previous results, the biotin-coupled miR-223-3p was better at capturing hsa_circ_0070963 in the complex as compared with the biotin-coupled NC (Figure 3E). Moreover, the product shown in Figure 3D was detected by qRT-PCR, followed by agarose gel electrophoresis (Figure 3F). This indicated that miR-223-3p could bind to hsa_circ_0070963. The sequences of the two binding regions between miR-223-3p and hsa_circ_0070963 are shown in Figure 3G. By using the pGL3-Basic construct, we generated a hsa_circ_0070963 luciferase reporter containing the miR-223-3p-binding sites (hsa_circ_0070963-Wt) or mutated sites (hsa_circ_0070963-Mu). The luciferase reporter. activity assays showed that the miR-223-3p mimic induced a decrease in the luciferase activity of the hsa_circ_0070963-Wt. In contrast, the miR-223-3p inhibitor caused an increase in hsa_circ_0070963-Wt luciferase activity (Figure 3H). However, both the miR-223-3p mimic and inhibitor showed no effects on hsa_circ_0070963-Mu luciferase activity (Figure 3H). In conclusion, these observations suggest that miR-223-3p is a direct target of hsa_circ_0070963.

### MiR-223-3p is related to the effects of hsa_circ_0070963 on HSC activation

**Figure 4:**
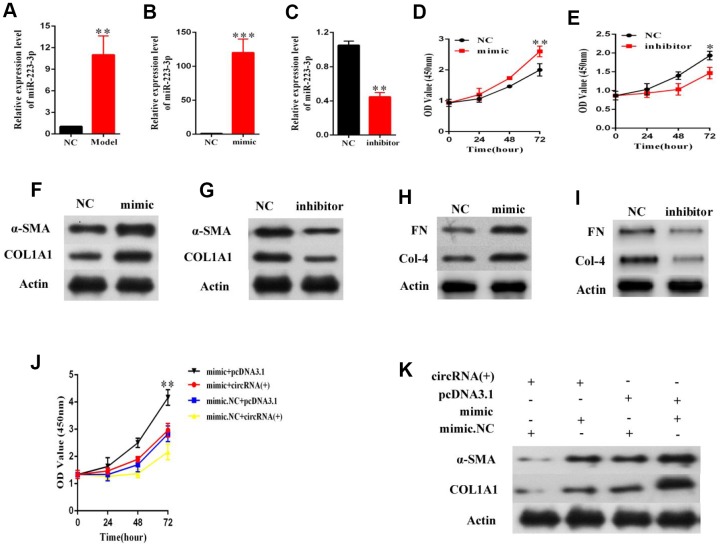
**MiR-223-3p is involved in the effects of hsa_circ_0070963 on HSCs activation.** (**A**) MiR-223-3p was analyzed in primary HSCs isolated from NC or CCl_4_-treated mice. The mRNA expression of miR-223-3p in HSCs was detected by qRT-PCR after transfection with a miR-223-3p mimic (**B**) or inhibitor (**C**). (**D**, **E**) Evaluation of cell proliferation LX2 cells transfected with a miR-223-3p mimic (**D**) or inhibitor (**E**) using the CCK-8 assay. (**F**, **G**) α-SMA and Col1A1 protein levels in LX2 cells transfected with a miR-223-3p mimic (**F**) or inhibitor (**G**) were measured by western blot. (**H**, **I**) FN and Col4 protein levels in LX2 cells transfected with a miR-223-3p mimic (**H**) or inhibitor (**I**) were quantified by western blot. (**J**) MiR-223-3p reversed the inhibitory effect of hsa_circ_0070963 on cell proliferation, as shown by the CCK-8 assay. (**K**) MiR-223-3p rescued the downregulation of α-SMA and ColA1 induced by hsa_circ_0070963 overexpression. Data are presented as means ± SD of three experiments (^*^p < 0.05, ^**^p < 0.01, and ^***^p < 0.001).

Considering the interaction between hsa_circ_0070963 and miR-223-3p, we next explored the potential function of miR-223-3p in HSCs. MiR-223-3p levels were found to be significantly increased in primary HSCs isolated from the fibrotic liver after CCl_4_ treatment (Figure 4A). Then, LX2 cells were transduced with a miR-223-3p mimic or inhibitor. As shown in Figure 4B and 4C, the qRT-PCR analysis confirmed a significant increase or suppression of miR-223-3p following treatment with the miR-223-3p mimic or inhibitor, respectively. Data from the CCK-8 assay suggested that miR-223-3p overexpression significantly promoted the proliferation of HSCs (Figure 4D), while decreased expression of miR-223-3p inhibited HSC proliferation (Figure 4E). Western blot experiments showed that overexpression or suppression of miR-223-3p increased or decreased α-SMA and ColA1 protein levels, respectively (Figure 4F, 4G). Additionally, the level of other proteins, such as FN and Col4, were also tested (Figure 4H, 4I). Rescue experiments were conducted by co-transfection of hsa_circ_0070963 and a miR-223-3p mimic in LX2 cells to assess whether the anti-fibrotic effect of hsa_circ_0070963 could be repressed by miR-223-3p overexpression. As expected, the miR-223-3p mimic could partly attenuate the inhibition of proliferation in LX2 cells caused by hsa_circ_0070963 overexpression (Figure 4J). Additionally, the miR-223-3p mimic could also rescue the reduction in α-SMA and ColA1 levels induced by hsa_circ_0070963 overexpression (Figure 4K). As such, these results indicated that miR-223-3p may be involved in the effects of hsa_circ_0070963 on HSC activation.

### LEMD3 is the target of miR-223-3p

**Figure 5:**
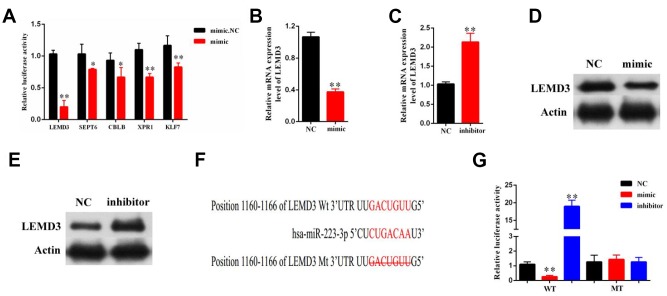
**LEMD3 is the target gene of miR-223-3p.** LX2 cells were transduced with a miR-223-3p mimic or inhibitor for 48 h. (**A**) The luciferase activity of 5 predicted mRNA targets in miR-223-3p mimic cells were examined by luciferase assay. (**B**, **C**) The mRNA expression of LEMD3 in the LX2 cell line was detected by qRT-PCR. (**D**, **E**) The protein expression of LEMD3 in the LX2 cell line was detected by western blot. (**F**) Predicted binding sites of miR-223-3p in the 3′-UTR of LEMD3. The mutated LEMD3 3′-UTR sequence is presented. (**G**) Relative luciferase activity was determined at 48 h after transfection with a miR-223-3p NC/mimic/inhibitor or LEMD3-Wt/Mu in HEK293T cells. Data are presented as means ± SD of three experiments (^*^p < 0.05 and ^**^p < 0.01).

Bioinformatics analysis revealed that LEMD3, SEPT6, CBLB, XPR1, and KLF7 were putative targets of miR-223-3p. We found that the luciferase activity of LEMD3 was the most significantly decreased in miR-223-3p mimic cells (Figure 5A). As such, this gene was chosen for further study. The qRT-PCR results indicated that LEMD3 expression was significantly decreased or increased in LX2 cells after treatment with a miR-223-3p mimic or inhibitor, respectively (Figure 5B, 5C). Moreover, miR-223-3p overexpression led to the reduction of LEDM3 protein levels (Figure 5D), while suppression of miR-223-3p increased LEMD3 levels (Figure 5E), as evident from the western blot data. In addition, we carried out luciferase reporter assays. The wild-type 3′-UTR sequence and the mutant 3′-UTR sequence of LEMD3 were cloned to construct reporter plasmids (Figure 5F). We noticed that co-transfection of the LEMD3-Wt reporter plasmids and a miR-223-3p mimic or inhibitor predominantly reduced or increased the luciferase activity, respectively. Conversely, co-transfection of LEMD3-Mu reporter plasmids and a miR-223-3p mimic or inhibitor showed no obvious effect on the luciferase activity (Figure 5G). These results indicate that LEMD3 is a target of miR-223-3p.

### Hsa_circ_0070963 inhibits HSC activation via miR-223-3p-mediated LEMD3 regulation

**Figure 6:**
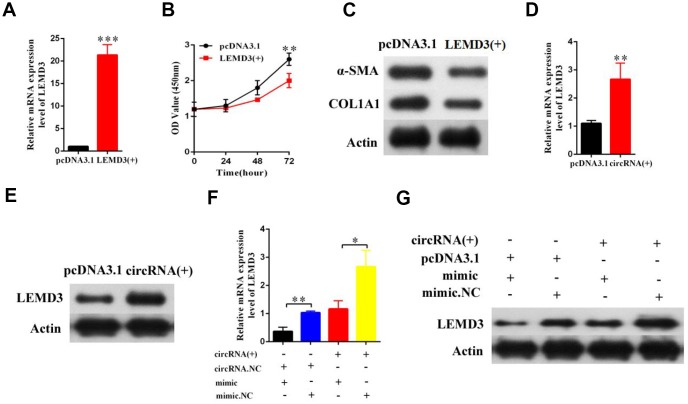
**Hsa_circ_0070963 modulates the expression of the endogenous miR-223-3p target LEMD3.** (**A**–**C**) LX2 cells were transduced with LEMD3 for 48 h. LEMD3 expression was detected by qRT-PCR (**A**) and cell proliferation was monitored using the CCK-8 assay (**B**). Then, the protein levels of α-SMA and Col1A1 were measured by western blot (**C**). (**D**, **E**) LX2 cells were transfected with hsa_circ_0070963 and the mRNA and protein expression levels of LEMD3 were determined by qRT-PCR (**E**) and western blot (**E**), respectively. (**F**) The results of the qRT-PCR showed that miR-223-3p could partly decrease the protein expression level of LEMD3 which were promoted by hsa_circ_0070963. (**G**) The western blot analysis showed that miR-223-3p could partly decrease the protein expression level of LEMD3 which were promoted by hsa_circ_0070963. Data are presented as means ± SD of three experiments (^*^p < 0.05, ^**^p < 0.01, and ^***^p < 0.001).

Next, we further sought to determine whether LEMD3 was involved in the effects of the hsa_circ_0070963-miR-223-3p axis on liver fibrosis. Enhanced expression of LEMD3 was confirmed by qRT-PCR (Figure 6A). The CCK-8 assay showed that overexpression of LEMD3 repressed proliferation in HSCs (Figure 6B). Notably, α-SMA and ColA1 were also inhibited by LEMD3 overexpression (Figure 6C). The mRNA and protein levels of LEMD3 were induced by hsa_circ_0070963 upregulation (Figure 6D, 6E). Moreover, we performed a co-transfection hsa_circ_0070963 and miR-223-3p to determine their combined effects on LEMD3 expression. Our results showed that miR-223-3p could partly suppress the promotion effect of hsa_circ_0070963 on the LEMD3 mRNA levels (Figure 6F), which was consistent with the results obtained when determining protein levels (Figure 6G). Altogether, these results indicate that hsa_circ_0070963 inhibits HSC activation via the miR-223-3p-LEMD3 axis.

### Hsa_circ_0070963 functions as a potential suppressor in liver fibrosis

**Figure 7:**
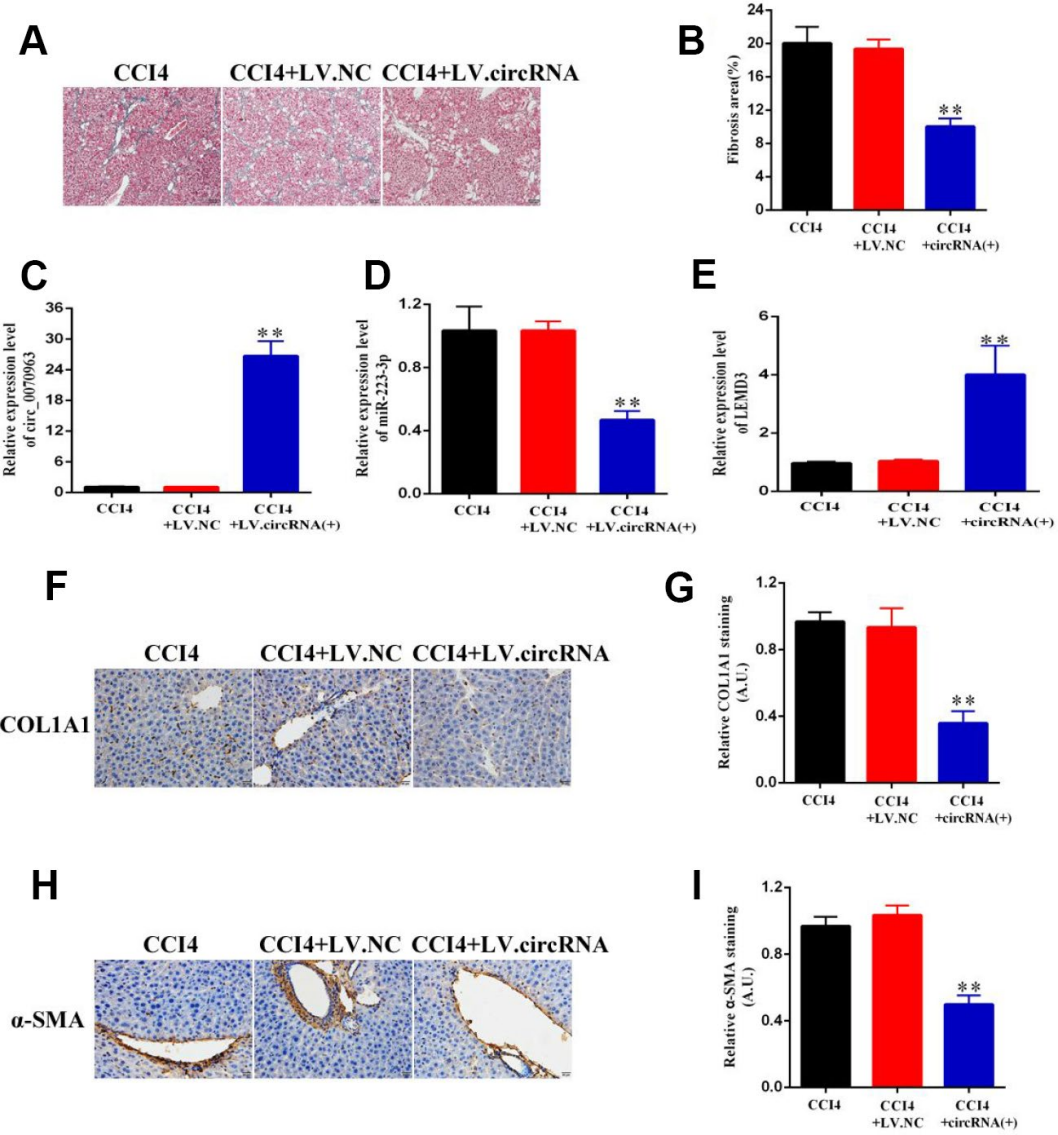
**Hsa_circ_0070963 upregulation suppresses CCl_4_-induced liver fibrosis in mice.** (**A**, **B**) Accumulation of collagen was assessed by Masson staining and the fibrosis area was quantified. The scale bar represents 100 μm. (**C**, **E**) The mRNA expression levels of hsa_circ_0070963 (**C**), miR-223-3p (**D**), and LEMD3 (**E**) were analyzed by qRT-PCR in each group. (**F**–**I**) Col1A1 and α-SMA levels were examined by immunohistochemistry and the results were quantified. The scale bar represents 100 μm. Data are presented as means ± SD of three experiments (^*^p < 0.05 and ^**^p < 0.01).

Finally, to better understand the role of hsa_circ_0070963 in the progression of liver fibrosis, lenti-hsa_circ_0070963 was injected into CCl_4_-treated mice via the tail vein to promote hsa_circ_0070963 expression. As illustrated by Masson staining (Figure 7A, 7B), hsa_circ_0070963 overexpression inhibited the accumulation of collagen caused by CCl_4_ treatment. As shown by the qRT-PCR analysis, administration of lenti-hsa_circ_0070963 greatly decreased miR-223-3p and increased LEMD3 levels (Figure 7C–7E) *in vivo*. Moreover, hsa_circ_0070963 upregulation led to the suppression of CCl_4_-induced α-SMA and Col1A1 levels (Figure 7E–7I). Therefore, our results demonstrate that upregulation of hsa_circ_0070963 attenuates CCl_4_-induced liver fibrosis *in vivo*.

## DISCUSSION

Liver fibrosis is a reversible wound-healing process designed to maintain organ integrity and is a pivotal precursor phase in the development of cirrhosis, which can ultimately lead to hepatocellular carcinoma without a liver transplant [22]. Although many drugs have been shown to have significant anti-fibrotic activity in mouse models, none of them appeared to be efficacious in the clinic. After being exposed to various factors, including viral infections, schistosomiasis, and alcoholism, HSCs are activated, which is associated with the increased production of excessive ECM components, such as α-SMA and collagens, eventually developing to liver fibrosis [23]. In the present study, we demonstrated that a circRNA, named hsa_circ_0070963, may inhibit liver fibrosis by sponging miR-223-3p and targeting LEMD3. Therefore, hsa_circ_0070963 may be a promising novel target and predictive marker for liver fibrosis.

As a novel domain of interest in the ncRNA field, circRNAs have been recently found to be associated with human disease and epigenetics. Previous research has shown that circRNAs have several advantages, such as stability, conservation, and tissue specificity [24]. The characteristics of circRNAs make them become promising therapeutic targets and potential biomarkers. Nevertheless, the role of circRNAs in liver fibrosis has rarely been reported. Recently, Zhu et al [25] showed that circRNA-0067835 acts as a sponge of miR-155 and accelerates FOXO3a expression to regulate the development of liver fibrosis. However, there are still a large number of circRNAs involved in liver fibrosis that have yet to be studied. In our previous work [14], we screened differentially expressed circRNAs in liver fibrosis by using circRNA microarrays and found that the expression of 179 circRNAs was increased and of 630 circRNAs was decreased by ≥2 fold between irradiated and control HSCs. Moreover, we observed that hsa_circ_0070963 was in the top 10 downregulated circRNAs in irradiated HSCs compared with normal HSCs. As such, in this study, we investigated the specific biological role of hsa_circ_0070963, which is transcribed from SCLT1 (Sodium channel and clathrin linker 1) on chromosome 4, in liver fibrosis. Our results showed a remarkable decrease of hsa_circ_0070963 expression in primary HSCs derived from the CCl_4_-treated mice when compared with the control mice. After Sal B treatment, which was reported to inhibit activated HSCs. including cell proliferation and collagen production [19], we observed that hsa_circ_0070963 levels were increased. *In vitro* functional experiments showed that the overexpression of hsa_circ_0070963 could inhibit HSC activation by inducing G1/S cell cycle arrest, cell growth inhibition, and a decrease in α-SMA and ColA1 levels. Similarly, *in vivo* experiments also confirmed the anti-fibrotic role of hsa_circ_0070963. Furthermore, evidence for hsa_circ_0070963 functionality in liver fibrosis was also shown to involve regulation of the miR-223-3p-LEMD3 axis. As far as we know, this is the first study to indicate that hsa_circ_0070963 plays an inhibitory role in liver fibrosis.

Previous research has shown that miR-223-3p inhibitors have a protective role against hypoxia-induced injuries by suppressing cardiomyocyte apoptosis and oxidative stress via KLF15 targeting. This indicates that suppression of miR-223-3p is likely a promising therapeutic target [26]. Another study showed that the expression of IL-6 and the phagocytic oxidase p47^phox^ was upregulated in miR-223-3p-knockout mice, resulting in ROS production, neutrophil infiltration, and hepatic injury after ethanol administration [27]. In our study, bioinformatics analysis was used to screen potential interactors and finally to confirm that miR-223-3p was the most likely to bind hsa_circ_0070963. Notably, miR-223-3p levels were obviously upregulated during liver fibrosis. Further functional studies verified that miR-223-3p promoted liver fibrosis progression and could partly block the hsa_circ_0070963-mediated inhibition of HSC activation. Thus, we showed that miR-223-3p was a direct target of hsa_circ_0070963 and that the latter may play a crucial role via the sponge activity of the former.

LEMD3 (also known as MAN1), is an inner nuclear membrane protein. A previous study has reported that loss-of-function mutations in *LEMD3* contributed to osteopoikilosis, Buschke-Ollendorff syndrome, and melorheostosis [28]. They also showed that LEMD3 could interact with BMP and TGF-β receptor-activated Smads and antagonized both signaling pathways in cells. In this study, LEMD3 was confirmed as a target of miR-223-3p. Overexpression of LEMD3 could inhibit HSC proliferation and diminish α-SMA and ColA1 protein levels, indicating that LEMD3 plays a role in HSC activation. Moreover, we found that hsa_circ_0070963 induced LEMD3 expression by sponging miR-223-3p in HSCs. Finally, we demonstrated that hsa_circ_0070963 inhibited CCl_4_-induced liver fibrosis in mice by modulating the miR-223-3p/LEMD3 axis. However, the mechanism underlying the regulation of hsa_circ_0070963 in liver fibrosis and interactions with other molecules need further verification and analysis.

Our current study demonstrates that hsa_circ_0070963 plays a role in the progression of liver fibrosis by acting as a miRNA sponge for miR-223-3p and thereby promoting the function of LEMD3. Our results illuminate a novel hsa_circ_0070963/miR-223-3p/LEMD3 signaling cascade in liver fibrosis, suggesting that hsa_circ_0070963 may be a candidate anti-fibrotic target.

## MATERIALS AND METHODS

### Plasmids transfection and reagents

Hsa_circ_0070963 and LEMD3 were cloned into the pcDNA3.1 vector (Geneseed Biotechnology Co., Guangzhou, China). The negative control (miR-NC), miR-223-3p mimic, and miR-223-3p inhibitor were designed and synthesized from GenePharma (Shanghai, China). Primary HSCs were transfected with the aforementioned constructs using Lipofectamine 2000 (Invitrogen, Carlsbad, CA, USA) at a final concentration of 100 nM according to the manufacturer’s instructions. At 48 h post-transfection, cells were collected for further experiments. The sequences of miR-223-3p mimic and inhibitor were as follows: miR-223-3p mimic: 5′-UGUCAGUUUGUCAAAUACCCA-3′; miR-223-3p inhibitor: 5**′**-UGGGGUAUUUGACAAACUGACA-3′.

CCl_4_ was purchased from Sigma-Aldrich (St. Louis, MO, USA). Salvianolic acid B (Sal B) was prepared as previously described [29].

### Cell culture

The human HSC cell line LX2 was purchased from ATCC and cultured in DMEM (Hyclone, Logan, UT, USA)‎ supplemented with 1% FBS (Gibco, Grand Island, NY, USA) and 1% penicillin-streptomycin. Primary HSCs were obtained from C57BL/6J mice as previously described [30]. The isolated cells were cultured in DMEM supplemented with 10% FBS and 1% penicillin-streptomycin. All cells were maintained in a 5% CO_2_ incubator at 37 °C. To confirm the culture purity, α-SMA immunocytochemical staining was performed and the purity was confirmed to reach > 98%.

### Quantitative real-time PCR (qRT-PCR)

Total RNA was extracted from primary HSCs using the TRIzol reagent (Invitrogen). Next, complementary DNA was synthesized using an SYBR Premix Ex Taq II kit (TaKaRa, Shiga, Japan). mRNA expression was monitored by real-time PCR using the SYBR-Green qPCR Master Mix (Toyobo, Osaka, Japan). The primer sequences are listed in Table 1.

**Table 1:** Primers used.

Has_circ_0070963-F	GTGAATGGAACCTCGCAGTC
Has_circ_0070963-R	AGTGTTTTGGCCTTGACAGA
miR-223-3p-F	GCAGAGTGTCAGTTTGTCAAAT
miR-223-3p-R	GCAGAGCGTGTATTTGACAAG
LEMD3-F	AACAAGACGCGGAACAGTAAT
LEMD3-R	GAGTCCGTAAGTAGGAGAGGTC
α-SMA-F	GTCCCAGACATCAGGGAGTAA
α-SMA-R	TCGGATACTTCAGCGTCAGGA
COL1-F	GAGGGCCAAGACGAAGACATC
COL1-R	CAGATCACGTCATCGCACAAC
Actin-F	CATGTACGTTGCTATCCAGGC
Actin-R	CTCCTTAATGTCACGCACGAT

### Western blot

To obtain total protein, HSCs were collected and lysed using the RIPA lysis buffer (Beyotime, China). Protein extracts were quantified and separated by SDS-PAGE. Then, western blot analysis was performed as previously described [30]. Antibodies against α-SMA, Col1A1, and β-Actin were purchased from Abcam (Cambridge, MA, USA). The anti-LEMD3 antibody was obtained from Santa Cruz (Dallas, TX, USA). All antibodies were diluted at 1:1000 in this experiment. Horseradish peroxidase (HRP)-conjugated secondary antibodies ((Millipore, Billerica, MA, USA) were used to detect protein bands.

### Flow cytometry analysis of the cell cycle

Cells were harvested and washed twice with PBS, then fixed with 70% ethanol at −20 °C for 24 h. Afterwards, cells were washed trice with PBS, resuspended in a PI/RNase staining buffer (PI; BD Pharmingen, San Jose, USA) containing 50 ng/μL RNase A for 30 min at 37 °C, and then analyzed on a BD LSRII flow cytometer (BD Biosciences, San Diego, CA, USA). The results were analyzed using the FlowJo V10 software (Tree Star Inc., Ashland, OR, USA).

### Cell proliferation analysis

To assess cell proliferation, cells were seeded in 96-well plates at a density of 2×10^3^ cells per well and cultured for different time periods. Cell proliferation was assessed using the CCK-8 (Dojindo, Kumamoto, Japan) following the manufacturer’s instructions. The optical density (OD) was determined at 450 nm.

### CCl_4_-induced liver fibrosis model

Male C57BL/6J mice aged eight weeks received an intraperitoneal injection of 10% CCl_4_ in olive oil (7 μL/g/mouse) biweekly for six weeks. Similarly, the control mice were treated with olive oil by using the same method.

For the CCl_4_-induced mouse liver fibrosis model, all C57BL/6J mice were randomly divided into three groups: mice treated with CCl_4_ alone (n = 6), CCl_4_ in combination with an injection of pNL-EGFP/CMV/WPREU3-NC (CCl_4_+NC; n = 6), and CCl_4_ in combination with an injection of pNL-EGFP/CMV/WPREU3-hsa_circ_0070963 (CCl_4_+CircRNA; n = 6). Every two weeks, the lentivirus (1 × 10^9^ pfu/100 μL) was injected into the mice via the tail vein for a total of six weeks. All animals were provided by the Experimental Animal Center of Wenzhou Medical University. The animal experimental protocol was approved by the University Animal Care and Use Committee in The First Affiliated Hospital of Nanjing Medical University. At the end of the experiment, mice were sacrificed under anesthesia and the livers were obtained for further studies such as Masson staining and immunohistochemistry.

### Confocal microscopy

Cells were fixed with 4% paraformaldehyde and then permeabilized with 0.3% Triton X-100 for 30 min at room temperature (RT). Non-specific binding was blocked with 3% bovine serum albumin (BSA) in PBS for 2 h at RT. Then, cells were incubated with the primary antibodies against α-SMA (dilution 1:300; Abcam) and Col1A1 (dilution 1:500; Abcam), overnight. Afterwards, cells were washed with PBS, followed by incubation with a FITC-conjugated secondary antibody (dilution 1:200; Invitrogen) for 2 h at RT in the dark. The cell nuclei were stained with 4,6-diamidino-2-phenylindole (DAPI; Molecular Probes, Eugene, OR, USA). The slides were analyzed using a laser scanning confocal microscope (Nikon, Nagoya, Japan).

### RNase R treatment

Total RNA (2 μg) was incubated for 30 min at 37 °C with 3 U/μg of RNase R (Epicentre Technologies, Madison, WI, USA). After treatment with RNase R, the RNA expression level of hsa-circ-0070963 was detected by Northern blot.

### 5-Ethynyl-20-deoxyuridine (EdU) incorporation assay

The EdU assay was performed using a Cell-Light EdU DNA Cell Proliferation Kit (RiboBio, Shanghai, PR, China). Briefly, approximately 1 × 10^4^ cells were seeded in a 96-well plate. After incubation with 50 mM EdU for 2 h, the cells were fixed and stained with Apollo Dye Solution. The cell nuclei were stained with DAPI. Images were acquired with a laser scanning confocal microscope, and the percentage of EdU-positive cells was calculated.

### Immunohistochemistry

Immunohistochemical staining was carried out as previously described [30]. Briefly, after dewaxing, hydration, and antigen retrieval, the liver sections were treated with primary antibodies against a-SMA (dilution 1:100; Abcam) or ColA1 (dilution 1:100; Abcam) overnight at 4 °C. The sections were then incubated with biotinylated anti-IgG (Vector Laboratories, Inc) secondary antibodies and detected using the avidin-biotin-peroxidase (ABC-Elite, Vector Laboratories, Burlingame, CA). The reaction products were visualized by diaminobenzidine (DAB) staining. Slides were counterstained with hematoxylin prior to desiccation and monitored using a microscope.

### Biotin-coupled probe pull-down assay

The pull-down assay was carried out by using a biotinylated RNA probe. Briefly, for hsa_circ_0070963 pulled down miRNAs, the biotinylated-hsa_circ_0070963 probe was bound to C-1 magnetic beads (Life Technologies, Carlsbad, CA, USA), then incubated with sonicated HSCs at 4 °C, followed by elution and detection via qRT-PCR. For miR-223-3p pulled down circRNAs, hsa_circ_0070963-overexpressing HSCs were transfected with a biotinylated miR-223-3p mimic. The cells were then lysed, sonicated, and incubated with C-1 magnetic beads, followed by elution and detection via qRT-PCR.

### Luciferase activity assay

The HEK293T cells were co-transduced with a luciferase reporter vector (pGL3-Basic-hsa_circ_0070963-Wt/-Mu or pGL3-Basic-3′-UTR-LEMD3-Wt/-Mu) and the miR-223-3p (NC/mimic/inhibitor) ectopic expression vector using Lipofectamine 2000. At 48 h post-transfection, cells were harvested and the luciferase activity was quantified using the dual-luciferase reporter assay system (Promega, Madison, WI, USA). The results are presented as the luminescence of the ratio of luciferase to renilla.

### Statistical analysis

The data from at least three independent experiments were presented as mean ± Standard Deviation (SD). Data were analyzed using either Student’s *t*-test (two-group comparison) or one-way *ANOVA* (more than two groups). A *p*-value of < 0.05 was considered statistically significant (^*^p < 0.05; ^**^p < 0.01; ^***^ *p* < 0.001). All graphs were made using the GraphPad Prism 7 software (GraphPad Software Inc., La Jolla, CA, USA).
